# Reconstruction of midface defects using local flaps: An algorithm for appropriate flap choice

**DOI:** 10.1097/MD.0000000000018021

**Published:** 2019-11-15

**Authors:** Jung Woo Chang, Jung Han Lim, Jang Hyun Lee

**Affiliations:** Department of Plastic and Reconstructive Surgery, Hanyang University Guri Hospital, Hanyang University College of Medicine, Guri, South Korea.

**Keywords:** advancement flap, algorithm, midface defect, transposition flap

## Abstract

**Background:**

Local flap surgery is commonly performed to cover defects with appropriate skin color and texture match. The purpose of this study was to present an algorithm for choosing an appropriate flap when reconstructing a midface defect using a local flap.

**Methods:**

Between February 2013 and February 2019, 38 patients with midface defects underwent local flap surgery. All defects larger than 3 cm in diameter were reconstructed with perforator-based transposition flaps. Defects smaller than 3 cm in diameter were reconstructed differently depending on their location. Defects near the nasolabial fold (NLF) were reconstructed with perforator-based transposition flaps, whereas defects just on the NLF were reconstructed with VY advancement flaps. Defects distant from the NLF were also reconstructed with VY advancement flaps.

**Results:**

Perforator-based transposition flaps were used in 22 cases and VY advancement flaps were used in 16 cases according to our new algorithm. All flaps survived without any complications. The aesthetic results were superior for VY advancement flaps, with higher patient satisfaction scores. The skin color match was similar for both flaps, but the contour was more natural in advancement flaps than in transposition flaps. However, transposition flaps had the benefits of being able to cover relatively large defects and allowing the donor scar to be hidden in a wrinkle line.

**Conclusion:**

The most suitable local flap for coverage of a midface defect can be chosen based on the patient's condition. By following our algorithm, appropriate reconstructions can be performed, with satisfactory results.

## 1 Introduction

The midface is an area defined by borders composed of the infraorbital rim, nasolabial fold (NLF), mandible lower border, and preauricular crease.^[1]^ Defects in this area result from various causes, such as neoplasm excision or trauma.^[2,3]^ As midface is most prominent and perceptible part of face, reconstructing midface defect requires both functional and esthetic outcomes. To cover such defects, many reconstructive options, such as primary closure, skin grafts, and local or free flaps, have been used.^[4–6]^ The options are usually chosen due to the size of defect. Free flap cannot be inevitable for large defect and primary closure is a suitable option for small defect enough to be closed without tension. However, most of midface defect show moderate size which cannot be closed primarily. For this reason, local flap and skin graft are widely used options for covering midface defect.^[7,8]^ Among these, local flaps show superior results in terms of color and contour match compared to skin grafts, which is why local flaps are generally preferred to cover midface defects.^[9]^

Local flaps can be divided into advancement flaps and transposition flaps according to the method of transfer.^[10,11]^ For covering midface defects, the VY advancement flap and perforator-based transposition flap are popular options.^[12,13]^ Both flaps are island flaps in terms of their shape, but the difference is the method of transfer. VY advancement flap is a method which advances V-shaped island flap to the defect resulting in Y shape as a final result. Otherwise, perforator-based transposition flap is a method which rotates an island flap to the defect with a pivot point of perforator pedicle. Both flaps provide advantages, but also have drawbacks. The choice of flap to use often depends on the surgeon's preference, which tends not to correspond to any systematic indication. An inappropriate choice of flap can result in an unacceptable outcome. The purpose of this study was to present an algorithm for choosing an appropriate flap when reconstructing a midface defect using a local flap.

## 2 Methods

**Figure 1:**
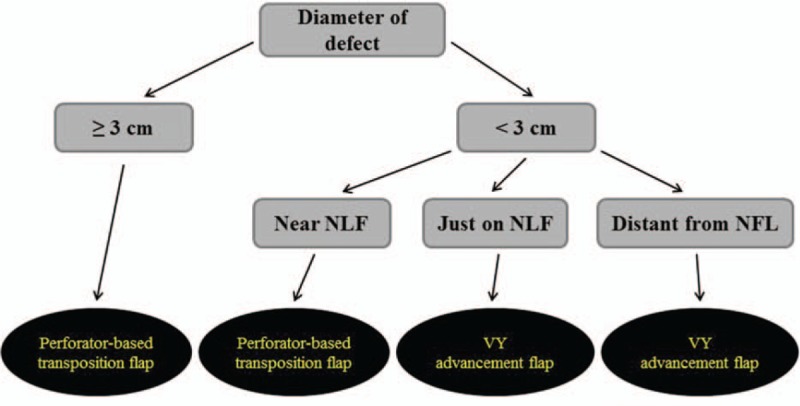
The new algorithm for flap choice when reconstructing a midface defect using a local flap. NLF = nasolabial fold.

**Figure 2:**
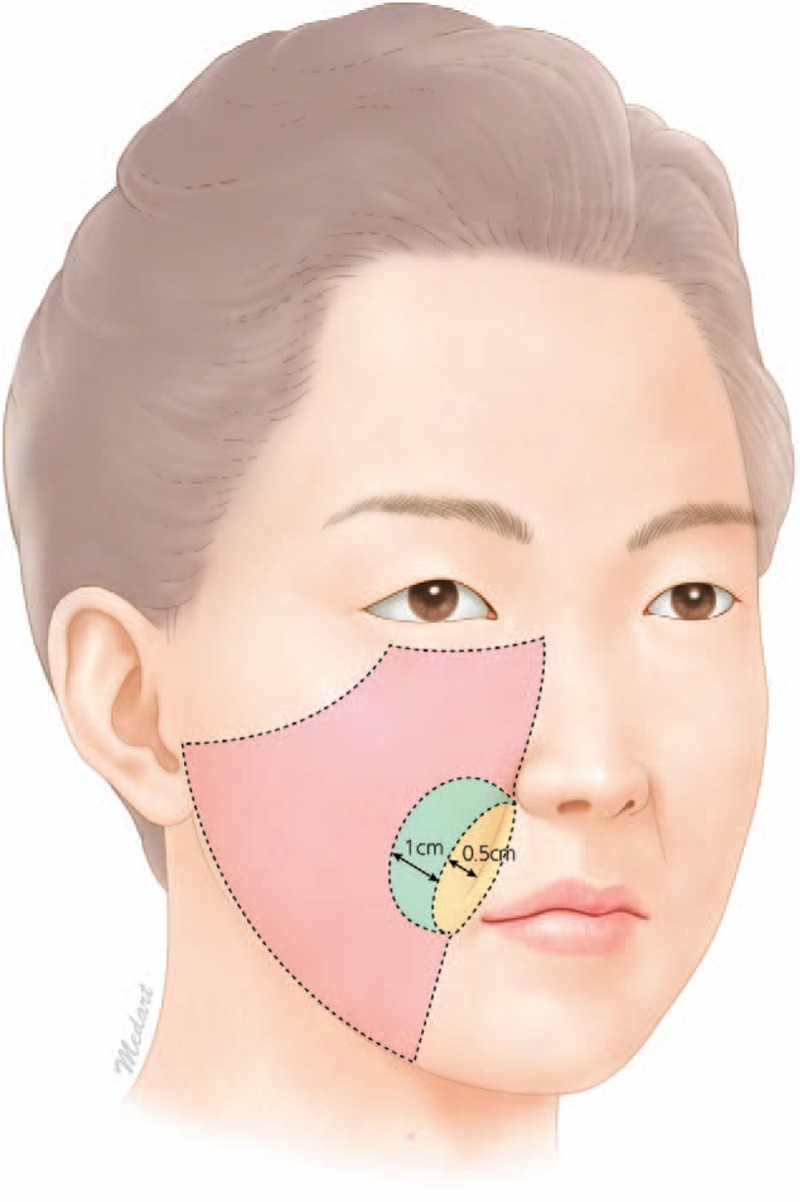
Illustration of the cheek area. The yellow color indicates the area just on the nasolabial fold (NLF) (<0.5 cm), the blue color indicates the area near the NLF (0.5–1.5 cm), and the pink color indicates the area distant from the NLF (>1.5 cm). NLF = nasolabial fold.

This study was conducted in conformity with the World Medical Association Declaration of Helsinki, and the protocol was approved by the Institutional Review Board of our institution (2018–07-023). From February 2013 to February 2019, 38 patients with a midface defect underwent local flap surgery. The choice between a VY advancement flap and a perforator-based transposition flap as the local flap was made following a new algorithm used in our clinic (Fig. 1). The diameter of the defect was measured, and the location of the defect was identified. If the diameter of the defect was larger than 3 cm, a perforator-based transposition flap was chosen for reconstruction regardless of the location. If the diameter of the defect was smaller than 3 cm, then the flap selection depended on the location of the defect. If a small defect was located near the NLF (i.e., if the center of a circular defect was located between 0.5 cm and 1.5 cm laterally from the midline of the NLF), a perforator-based transposition flap was chosen. If a small defect was located just on the NLF (i.e., if the center of a circular defect was located not farther than 0.5 cm laterally from the midline of the NLF), a VY advancement flap was chosen. If a small defect was distant from the NLF (i.e., if the center of a circular defect was located farther than 1.5 cm from the midline of the NLF), then a VY advancement flap was also chosen (Fig. 2).

When performing perforator-based transposition flap surgery, a perforator was marked near the defect with using hand-held Doppler. The length of the flap was determined by measuring the length from the distal edge of the defect to the perforator marking, which became a pivot point. The flap was designed to hide the donor site scar within the relaxed skin tension line as much as possible. The flap was elevated as an island type in a fasciocutaneous fashion and rotated to the defect. Each VY advancement flap was designed as a V-shape flap with a length-to-width ratio of 2:1. The flap was designed beside the defect, and elevated in a fasciocutaneous fashion. The flap base was dissected until the advancement of the flap to the defect without tension became possible.

**Figure 3:**
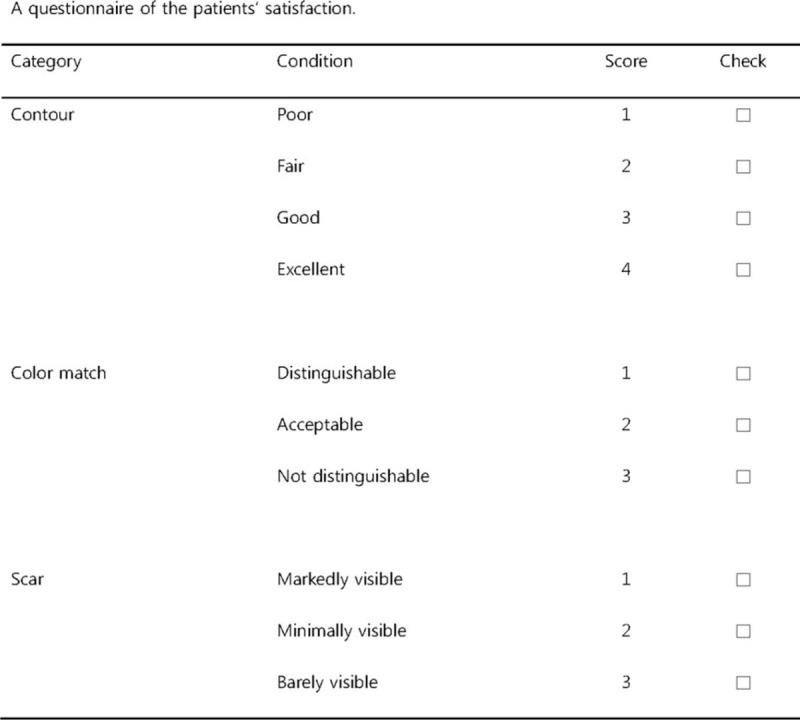
A questionnaire of the patients’ satisfaction score.

A retrospective review of the case notes was performed. Data were collected, including patients’ demographics, defect size, postoperative complications, and aesthetic results. Postoperative complications and aesthetic results were evaluated at 1 week and 6 months postoperatively. Complications, such as wound problems or flap loss, were assessed by clinical signs. The aesthetic results were assessed by the patients’ satisfaction using a questionnaire, on which a score of 10 points corresponded to the highest level of satisfaction (Fig. 3). Statistical analysis of the scores, with a comparison between the advancement flap group and the transposition flap group, was performed using SPSS version 20.0 (IBM Corp, Armonk, NY). *P* values ≤.05 were considered to indicate statistical significance.

## 3 Results

The total population of 38 patients consisted of 11 males and 27 females. Their mean age was 70.5 years (range, 49–90 years). Two groups were divided according to flap type, group I of VY advancement flaps were used in 22 cases, whereas group II of perforator-based transposition flaps were used in 16 cases. There is no difference for age between 2 groups but the defect size of group II is larger than that of group I (*P* ≤ .05) (Table 1).

**Table 1:**
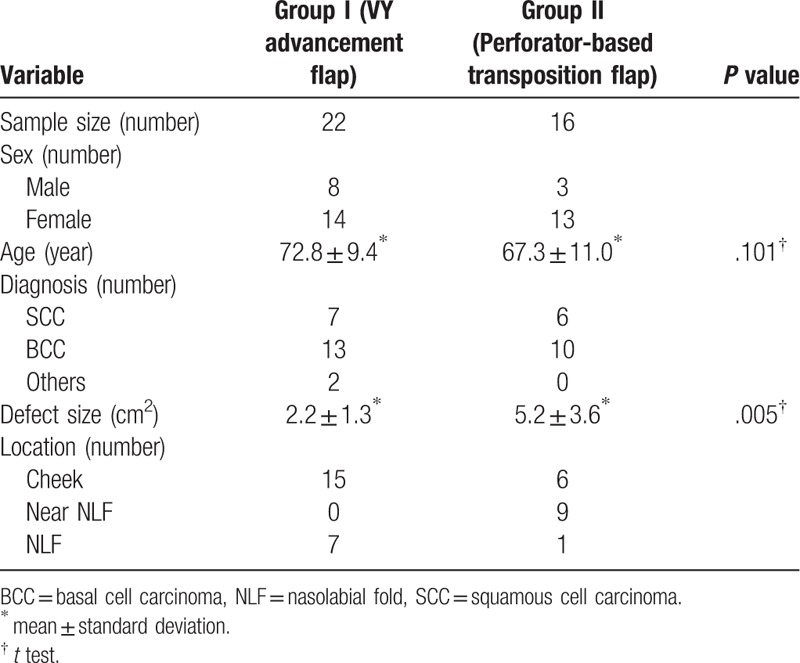
Demographic, diagnosis, and defect size and location by flap type.

Among the 38 cases, 7 involved a defect larger than 3 cm in diameter. These defects were all reconstructed with perforator-based transposition flaps. Thirty-one cases involved a defect smaller than 3 cm in diameter. Of those 31 cases, 9 cases with a defect near the NLF were reconstructed with perforator-based transposition flaps, 7 cases with a defect within the NLF were reconstructed with VY advancement flaps, and 15 cases with a defect distant from the NLF were reconstructed with VY advancement flaps.

There were no major complications, such as flap loss, in any cases. In 2 cases in which perforator-based transposition flaps were used, delayed wound healing was observed, but there was no necessity for a revisional procedure. The average of satisfaction score of a total of 38 patients increased during the follow-up period, from 6.9 at 1 week postoperatively to 8.1 at 6 months postoperatively. The patients who received a VY advancement flap consistently reported significantly higher scores than those who received a perforator-based transposition flap. The average scores of the 2 groups at 1 week postoperatively were 7.2 and 6.5 and the scores at 6 months were 8.4 and 7.8, respectively (*P* ≤ .05) (Table 2).

**Table 2:**
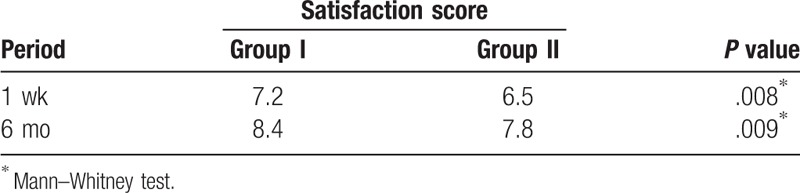
Comparison of the satisfaction scores between Group I (VY advancement flap) and Group II (perforator-based transposition flap).

## 4 Case 1

**Figure 4:**
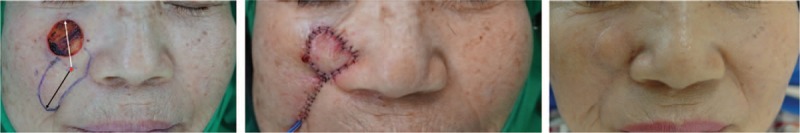
A case with a defect larger than 3 cm in diameter. A 76-year-old female patient with squamous cell carcinoma on the right cheek. (Left) The size of the defect after wide excision was 3.5 × 3.0 cm. For designing perforator-based transposition flap, a perforator was marked (red dot) and the length of the flap (black arrow) was determined by measuring the length from the distal edge of the defect to the perforator marking (white arrow). (Center) The defect was covered with a perforator-based transposition flap. (Right) The wound healed without any complications, but a mildly unnatural contour was seen in the early postoperative period.

A case with a defect larger than 3 cm in diameter on the cheek (Fig. 4). A 76-year-old female patient with squamous cell carcinoma (SCC) on her right cheek underwent wide excision. The size of the defect after wide excision was 3.5 × 3.0 cm, and the defect was covered with a perforator-based transposition flap. The wound healed without any complications, but a mildly unnatural contour was seen in the early postoperative period. The patient's satisfaction score was 7 at 1 week postoperatively and increased to 9 at 6 months postoperatively.

## 5 Case 2

**Figure 5:**
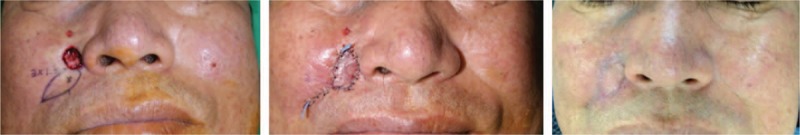
A case with a defect smaller than 3 cm in diameter near the NLF. A 62-year-old male patient with basal cell carcinoma near the right NLF. (Left) The size of the defect after wide excision was 1.0 × 1.0 cm. (Center) The defect was covered with a perforator-based transposition flap. (Right) The wound healed without any complications, but trapdoor deformity on the flap margin was observed in the early postoperative period. NLF = nasolabial fold.

A case with a defect smaller than 3 cm in diameter near the NLF (Fig. 5). A 62-year-old male patient with basal cell carcinoma (BCC) near the right NLF underwent wide excision. The size of the defect after wide excision was 1.0 × 1.0 cm, and a perforator-based transposition flap was chosen for defect coverage. No complications were observed during the follow-up period. The donor scar was completely hidden within the NLF, but a trapdoor deformity on the flap margin was observed in the early postoperative period. The patient's satisfaction score was 6 at 1 week postoperatively and 8 at 6 months postoperatively.

## 6 Case 3

**Figure 6:**
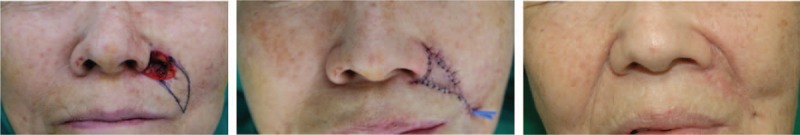
A case with a defect smaller than 3 cm in diameter just on the NLF. An 82-year-old female patient with basal cell carcinoma on the left NLF. (Left) The size of the defect after wide excision was 2.2 × 1.8 cm. (Center) The defect was covered with a VY advancement flap. (Right) The wound healed without any complications, and the contour of the flap was quite natural. NLF = nasolabial fold.

A case with a defect smaller than 3 cm in diameter just on the NLF (Fig. 6). An 82-year-old female patient with BCC on the left NLF underwent wide excision. The size of the defect after wide excision was 2.2 × 1.8 cm, and the defect was covered with a VY advancement flap. There were no complications, and the contour of the flap was quite natural. The patient's satisfaction score was 8 at 1 week postoperatively and 9 at 6 months postoperatively.

## 7 Case 4

**Figure 7:**
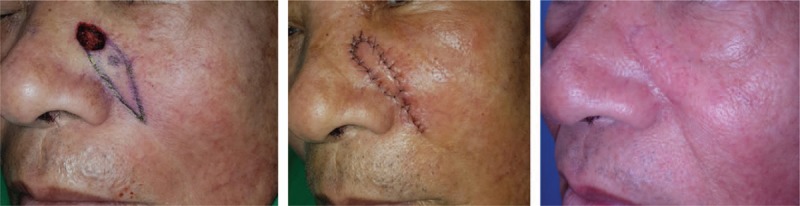
A case with a defect smaller than 3 cm in diameter distant from the nasolabial fold. A 70-year-old male patient with basal cell carcinoma on the left paranasal area. (Left) The size of the defect after wide excision was 1.0 × 0.9 cm. (Center) The defect was covered with a VY advancement flap. (Right) The wound healed without any complications, and the flap showed a natural contour.

A case with a defect smaller than 3 cm in diameter distant from the NLF (Fig. 7). A 70-year-old male patient with BCC on the left paranasal area underwent wide excision. The size of the defect after wide excision was 1.0 × 0.9 cm, and a VY advancement flap was used for defect coverage. The flap took without any complications, and showed a natural contour during the follow-up period. The patient's satisfaction score was 8 at 1 week postoperatively and increased to 9 at 6 months postoperatively.

## 8 Discussion

**Figure 8:**
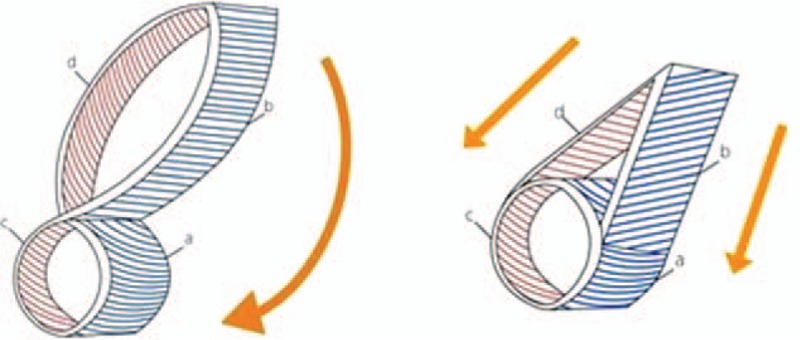
Schematic images of the relationship between the defect depth and the flap thickness. (Left) Perforator-based transposition flap. After rotating a flap, the thick side of the flap (B) is in contact with the shallow side of the defect (C), whereas the thin side of the flap (D) is in contact with the deep side of the defect (A). (Right) VY advancement flap. After advancing a flap, the thick side of the flap (B) is in contact with the deep side of the defect (A), and the thin side of the flap (D) is in contact with the shallow side of the defect (C).

Perforator-based island flaps can be designed in various ways based on the numerous perforators in the midface.^[14–16]^ As this flap covers a defect by rotation with a perforator as the pivot, a relatively large defect can be covered. In addition, low donor site morbidity is another advantage of this flap.^[17–19]^ However, the unnatural contour of the flap after covering a defect is a weak point.^[20–22]^ In this study, some patients treated with a perforator-based island flap showed unnatural contours, such as trapdoor deformity, as indicated by lower patient satisfaction scores. This contour problem may result from depth mismatch between the defect and the flap. After rotating a flap, the thick side of the flap is placed in contact with the shallow side of the defect, whereas the thin side of the flap is in contact with the deep side of the defect (Fig. 8). This mismatch provides an unnatural contour at the flap margin.

The VY advancement flap is a classical flap that was used before the concept of perforators emerged. However, this flap provides a more natural postoperative contour than the perforator-based island flap.^[23]^ After advancing a flap, the thick side of the flap is placed in contact with the deep side of the defect, and the thin side of the flap is in contact with the shallow side of the defect (Fig. 8). This results in a similar contour between the flap and the adjacent tissue after defect coverage. Despite the superior results of the contour, the advancement flap has a limitation in the length that a flap can be advanced to a defect, meaning that a defect with a relatively large diameter cannot be covered with this flap.^[24–27]^ Based on our experiences, we concluded that the maximal length of advancement for defect coverage without tension is 3 cm, and this cut-off was applied in the algorithm used in this study.

There is no difference in color match between the 2 flaps, because both local flaps are elevated near the defect. However, to maximize the advantages of both flaps, following our algorithm is beneficial. For covering a defect larger than 3 cm in diameter, using a perforator-based transposition flap is essential, as it can cover a large defect. Meanwhile, for covering a defect smaller than 3 cm in diameter, a VY advancement flap is suitable, as it results in a more natural contour. There is only one exceptional condition in covering a small defect. If a defect is located near the NLF, a perforator-based transposition flap is a better option regardless of defect size, because the donor site scar can be hidden within the NLF by designing a flap with 90° rotation.

## 9 Conclusion

For reconstructing a midface defect with a local flap, it is important to choose an appropriate flap for functional and aesthetic outcomes. Pursuing only a single option in every case is not suitable. The flap must be appropriately selected according to the defect size and location. By following our algorithm for reconstructing midface defects, satisfactory results can be obtained.

## Author contributions

**Conceptualization:** Jung Woo Chang, Jang Hyun Lee.

**Data curation:** Jung Han Lim.

**Formal analysis:** Jung Han Lim.

**Methodology:** Jang Hyun Lee.

**Supervision:** Jang Hyun Lee.

**Writing – original draft:** Jung Woo Chang, Jang Hyun Lee.

**Writing – review & editing:** Jung Woo Chang, Jung Han Lim, Jang Hyun Lee.

## References

[1] Pletcher SD Kim DW Current concepts in cheek reconstruction. Facial Plast Surg Clin North Am 2005;13:267–81. vi. 15817406 10.1016/j.fsc.2004.11.004

[2] Eskander A Kang SY Teknos TN Advances in midface reconstruction: beyond the reconstructive ladder. Curr Opin Otolaryngol Head Neck Surg 2017;25:422–30. 28692450 10.1097/MOO.0000000000000396

[3] Kawase-Koga Y Mori Y Saijo H Reconstruction of a complex midface defect from excision of a squamous cell carcinoma, according to regional aesthetic units. Oral Surg Oral Med Oral Pathol Oral Radiol 2014;117:e97–101. 24113351 10.1016/j.oooo.2013.06.038

[4] Chang EI Hanasono MM State-of-the-art reconstruction of midface and facial deformities. J Surg Oncol 2016;113:962–70. 27226161 10.1002/jso.24150

[5] Cho K-H Uthaman S Pakr I-K Injectable biomaterial in plastic and reconstructive surgery: a review of the current status. Tissue Eng Regen Med 2018;15:559–74. 30603579 10.1007/s13770-018-0158-2 PMC6171701

[6] Shan X Choi JH Kim KJ Adipose stem cells with conditioned media for treatmnet of acne vulagis scar. Tissue Eng Regen Med 2018;15:49–61. 30603534 10.1007/s13770-017-0105-7 PMC6171636

[7] Liu X Liu Y Chen K Reconstruction of skin defects in the medial cheek using lateral cheek rotation flap combined with Z-plasties. J Plastic Reconstr Aesthet Surg 2015;68:e183–8. 10.1016/j.bjps.2015.07.014 26243195

[8] Ozkaya Mutlu O Egemen O Dilber A Aesthetic unit-based reconstruction of periorbital defects. J Craniofac Surg 2016;27:429–32. 26963300 10.1097/SCS.0000000000002359

[9] Kim SW Kim YH Kim JT Angular artery perforator-based transposition flap for the reconstruction of midface defect. Int J Dermatol 2012;51:1366–70. 23067088 10.1111/j.1365-4632.2012.05516.x

[10] Pepper JP Baker SR Local flaps: cheek and lip reconstruction. JAMA Facial Plast Surg 2013;15:374–82. 24051684 10.1001/jamafacial.2013.1608

[11] Starkman SJ Williams CT Sherris DA Flap basics I: rotation and transposition flaps. Facial Plast Surg Clin North Am 2017;25:313–21. 28676159 10.1016/j.fsc.2017.03.004

[12] Jayarajan R A combination flap for nasal defect reconstruction. Ann Plast Surg 2018. 10.1097/SAP.0000000000001483 29781857

[13] Rossi M Milia A Carmisciano M Advancement perforator cheek flap for aesthetic one-stage reconstruction of postoncological extended split-thickness defects of the nasal sidewall. ScientificWorldJournal 2013. 10.1155/2013/169208 PMC3826316 24288460

[14] Iyer K Chen Z Ganapa T Keratinocyte migration in a three-dimensional in vitro wound healing model co-cultured with fibroblasts. Tissue Eng Regen Med 2018;15:721–33. 30603591 10.1007/s13770-018-0145-7 PMC6250652

[15] Qassemyar Q Havet E Sinna R Vascular basis of the facial artery perforator flap: analysis of 101 perforator territories. Plast Reconstr Surg 2012;129:421–9. 22286424 10.1097/PRS.0b013e31822b6771

[16] Yoon TH Yun IS Rha DK Reconstruction of various perinasal defects using facial artery perforator-based nasolabial island flaps. Arch Plast Surg 2013;40:754–60. 24286050 10.5999/aps.2013.40.6.754 PMC3840184

[17] D’Arpa S Cordova A Pirrello R Free style facial artery perforator flap for one stage reconstruction of the nasal ala. J Plast Reconstr Aesthet Surg 2009;62:36–42. 18945660 10.1016/j.bjps.2008.06.057

[18] Mann R Srinivasan B Webb R An unusual complication of nasolabial flap reconstruction. Ann R Coll Surg Engl 2017;99:e60–1. 27791423 10.1308/rcsann.2016.0332 PMC5392839

[19] Zhao XP Zhang H Yu X Reverse facial artery flap to reconstruct the medium-sized defects in the middle facial region following cancer ablation. J Craniofac Surg 2013;24:2077–81. 24220410 10.1097/SCS.0b013e3182a21100

[20] Cho K-H Singh B Maharjan S Local delivery of CTGF siRNA with Poly(sorbital-co-REI) reduces scar contraction in cutaneous wound healing. Tissue Eng Regen Med 2017;14:211–20. 30603478 10.1007/s13770-017-0059-9 PMC6171600

[21] Bi H Xing X Li J Nasolabial-alar crease: a natural line to facilitate transposition of the nasolabial flap for lower nasal reconstruction. Ann Plast Surg 2014;73:520–4. 25305185 10.1097/SAP.0b013e31827f547e

[22] El Habr C Vinelli G Tinklepaugh A Reconstruction of an alar defect with a fusiform nasolabial turnover flap with a proximal, superiorly tapered apex. J Craniofac Surg 2018;29:e20–1. 28968314 10.1097/SCS.0000000000003990

[23] Motamedi MH Behnia H Experience with regional flaps in the comprehensive treatment of maxillofacial soft-tissue injuries in war victims. J Craniomaxillofac Surg 1999;27:256–65. 10626260 10.1016/s1010-5182(99)80038-9

[24] Ryu YH Lee YJ Kim K-J Epidermal growth factor (EGF)-like repeats and discoidin I-like domains 3 (EDIL3): a potential new therapeutic tool for the treatment of keloid scars. Tissue Eng Regen Med 2017;14:267–77. 30603483 10.1007/s13770-017-0034-5 PMC6171597

[25] Boyette JR Vural E Cervicofacial advancement-rotation flap in midface reconstruction: forward or reverse? Otolaryngol Head Neck Surg 2011;144:196–200. 21493415 10.1177/0194599810391391

[26] Li JH Xing X Li P Transposition movement of V-Y flaps for facial reconstruction. J Plast Reconstr Aesthet Surg 2007;60:1244–7. 17950187 10.1016/j.bjps.2006.10.011

[27] Yildirim S Akoz T Akan M Nasolabial V-Y advancement for closure of the midface defects. Dermatol Surg 2001;27:656–8. 11442618 10.1046/j.1524-4725.2001.00346.x

